# A new model measuring bacterial phagocytosis and phagolysosomal oxidation in humans using the intradermal injection of methylene blue–labeled *Escherichia coli*

**DOI:** 10.1093/jleuko/qiae217

**Published:** 2024-10-16

**Authors:** George B Collins, Jhonatan de Souza Carvalho, Sandali C Jayasinghe, Urte Gumuliauskaite, David M Lowe, David C Thomas, Erik Årstad, Roel P H De Maeyer, Derek W Gilroy

**Affiliations:** Department of Ageing, Rheumatology and Regenerative Medicine, Division of Medicine, University College London, London WC1E 6JF, United Kingdom; Department of Cardiology, St Bartholomew's Hospital, Barts Health NHS Trust, London EC1A 7BE, United Kingdom; Department of Ageing, Rheumatology and Regenerative Medicine, Division of Medicine, University College London, London WC1E 6JF, United Kingdom; Department of Diagnosis and Surgery, School of Dentistry, São Paulo State University, São Paulo 14801-903, Brazil; Department of Ageing, Rheumatology and Regenerative Medicine, Division of Medicine, University College London, London WC1E 6JF, United Kingdom; Department of Ageing, Rheumatology and Regenerative Medicine, Division of Medicine, University College London, London WC1E 6JF, United Kingdom; Institute of Immunity and Transplantation, The Pears Building, University College London, London NW3 2PP, United Kingdom; Cambridge Institute of Therapeutic Immunology and Infectious Disease, Jeffrey Cheah Biomedical Centre, Cambridge Biomedical Campus, University of Cambridge, Cambridge CB2 0AW, United Kingdom; Centre for Radiopharmaceutical Chemistry, University College London, London WC1E 6BS, United Kingdom; Botnar Research Centre, Nuffield Department of Orthopaedics, Rheumatology and Musculoskeletal Medicine, University of Oxford, Oxford OX3 7LD, United Kingdom; Department of Ageing, Rheumatology and Regenerative Medicine, Division of Medicine, University College London, London WC1E 6JF, United Kingdom

**Keywords:** human challenge models, infection, neutrophils, phagocytosis, phagolysosomal oxidation

## Abstract

Phagocytosis is an important leukocyte function; however, using existing models it cannot be measured in human tissues in vivo. To address this, we characterized a new phagocytosis model using intradermal methylene blue–labeled *Escherichia coli* injection (MBEC). Methylene blue (MB) is a licensed human medicine and bacterial stain potentially useful for labeling *E. coli* that is safe for human injection. Ex vivo coculture of leukocytes with MBEC caused MB to transfer into neutrophils and macrophages by phagocytosis. During this, a “red shift” in MB fluorescence was shown to be caused by phagolysosomal oxidation. Hence, MBEC coculture could be used to measure phagocytosis and phagolysosomal oxidation in humans, ex vivo. In healthy volunteers, inflammatory exudate sampling using suction blisters 2 to 24 h after intradermal MBEC injection showed that tissue-acquired neutrophils and monocytes contained more MB than their circulating counterparts, whereas blood and inflamed tissue T, B, and natural killer cells were MB^lo^. This was validated with spectral flow cytometry by visualizing the MB emission spectrum in tissue-acquired neutrophils. Neutrophil MB emission spectra demonstrated more red shift at 24 h compared with earlier time points, in keeping with progressive phagolysosomal MB oxidation in neutrophils over time in vivo. This new MBEC model can therefore measure bacterial phagocytosis and phagolysosomal oxidation in human skin, in vivo. This has a number of important research applications, e.g. in studying human phagocyte biology, testing novel antimicrobials, and understanding why certain groups such as males, the elderly or those with diabetes, recent surgery, or malnutrition are at increased risk of bacterial infection.

## Teaser

This study characterized a new human challenge model to measure bacterial phagocytosis and phagolysosomal oxidation in human skin in vivo using the intradermal injection of methylene blue–labeled *Escherichia coli*, followed by inflammatory exudate sampling using suction blister formation and flow cytometry analysis of infiltrating leukocytes.

## 1. Introduction

Phagocytosis is an important leukocyte function; however, there are no established experimental models that can measure this process in human tissues, in vivo.^1–3^ This is important because tissues are the primary site of bacterial infection and phagocyte effector function, and many important phenotypic differences exist between circulating and tissue-acquired phagocytes.^4–9^ As a result, previous studies of bacterial phagocytosis are restricted to in silico studies, murine models, and ex vivo culture of fluorescently labeled particles with peripheral human blood leukocytes.^10–12^ However, being performed outside the in vivo human body, these studies have limited translational capacity, which is hampering progress in our understanding of in vivo human phagocyte biology.^13–19^

The intradermal injection of ultraviolet (UV) light–killed *Escherichia coli* in humans is an established skin challenge model that generates a robust and self-resolving inflammatory response and has provided important and novel insights into human in vivo immune responses.^20–24^ Sampling inflammatory exudates by suction blister formation and drainage at different time points after *E. coli* injection allows for temporal analysis of infiltrating leukocyte abundance and surface marker expression by flow cytometry. After human intradermal *E. coli* injection, professional phagocytes such as neutrophils and monocytes are the first and most abundant infiltrating leukocytes to migrate into the site of inflammation, followed by adaptive immune cells such as lymphocytes and natural killer cells.^20^ However, as the *E. coli* in this existing model are unlabeled, they cannot be used to measure phagocytosis in human skin, in vivo.

**Fig. 1.**
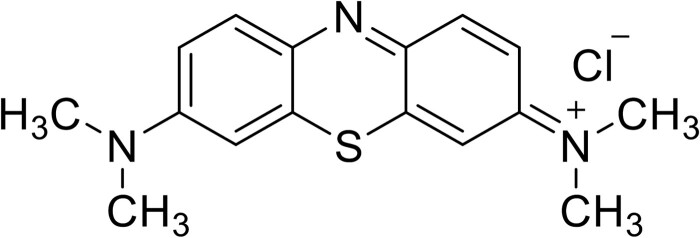
Chemical structure of MB (methylthioninium chloride).

Methylene blue (MB) (Fig. 1) is a licensed human medicine and established bacterial stain that could be used to label UV-killed *E. coli* that are safe for human injection. MB-labeled *E. coli* (MBEC) could therefore be used to measure phagocytosis in humans in vivo.^25–27^ Furthermore, because MB is a red-excited and red-emitting fluorophore, it is compatible with the detection range of both conventional and spectral flow cytometers.^28–30^ MBEC could therefore be used alongside conjugated antibody staining of infiltrating dermal leukocytes to simultaneously measure their surface marker expression and phagocytic function in humans, in vivo.

This new model could overcome many of the translational limitations of existing ex vivo and murine phagocytosis models, and therefore has a number of important potential research applications. On that basis, we set out to characterize a new model to measure bacterial phagocytosis in human skin in vivo using the intradermal injection of MBEC in healthy volunteers.

## 2. Methods

### 2.1 Ethics statement

For healthy volunteers, ethical approval was granted by the University College London Institutional Ethics Committee (ID: 1309/004), and for patients with chronic granulomatous disease (CGD) it was provided by the local Health Research Authority Research Ethics Committee (ID: 15/LO/1334). Volunteers provided written informed consent. All procedures were performed according to the 1975 Helsinki Declaration.

### 2.2 Recruitment

Unless otherwise stated, healthy, young (18 to 40 yr), nonsmoking volunteers of either sex and any ethnicity were recruited. Exclusion criteria were chronic inflammatory disease, recent illness, recent vaccination (≤3 mo), routine blood test abnormalities, and any medication taken in the preceding week. Volunteers were required to refrain from alcohol and heavy exercise during the study.

### 2.3 Methylene blue

For ex vivo experiments, MB hydrate (Sigma-Aldrich) was diluted in phosphate-buffered saline (PBS) (Gibco) to the indicated concentration and filtered (Whatman; 11 µm). For in vivo experiments, pharmaceutical grade 1% aqueous MB (methylthioninium chloride) was used instead (FlexiPharm Austrading). MB light absorbance was measured by spectrophotometry (Tecan Spark), and emission after red laser excitation during fluorescence microscopy (detection range 673 to 743 nm) and flow cytometry. Spectrophotometry was performed with or without hydrogen peroxide (LP Chemicals) at the indicated concentrations.

The sensitivity of flow cytometric detection of MB was increased (1) using above-default detector voltages (Alexa Fluor 647 [AF647] = 650, AF700 = 650, APC-Cy7 = 600) with conventional flow cytometry, (2) using the 637 nm detector at 70 V with spectral flow cytometry, and (3) by merging the AF647, AF700, and APC-Cy7 channels post hoc using FCS Express software version 7.22.0031 (De Novo). In conventional flow cytometry, a “red shift” in neutrophil MB fluorescence was visualized by dividing the APC-Cy7 channel (geometric) mean fluorescence intensity (MFI) by the AF647 channel MFI, and in spectral flow cytometry by dividing each data point on the MB emission curve by the sum of all data points for each condition. This corrects for differences in MB MFI between conditions and allows for side-by-side comparison of leukocyte MB emission spectra.

### 2.4 Preparation of human leukocytes and serum

Circulating leukocytes from the venous blood of healthy volunteers were sampled by venepuncture (Greiner Bio-One), anticoagulated with EDTA (BD), and isolated by diluting 1:10 with ACK lysis buffer (Gibco) for 6 min at room temperature (RT). Leukocytes were pelleted (500 *g*, 5 min, RT), washed in Hanks’ Balanced Salt Solution (Gibco), and resuspended in RPMI 1640 medium (Gibco) + 10% fetal calf serum (Gibco). To label leukocytes with MB, they were fixed in 2% PFA (Thermo Fisher Scientific), incubated in 1% MB in PBS for 60 min, and washed in Hanks’ Balanced Salt Solution (Gibco). Neutrophils and monocytes were isolated by magnetic bead negative selection according to manufacturer's instructions using the EasySep and RosetteSep Isolation kits, respectively (STEMCELL Technologies). Monocytes were matured into monocyte-derived macrophages using 7-d culture in recombinant human macrophage colony-stimulating factor (50 ng/mL; Sigma-Aldrich) at 37 °C, before harvesting with ice-cold Accutase enzymatic cell detachment media (Invitrogen). Autologous human serum was prepared from venous blood coagulated in serum separation tubes (BD) and isolated by centrifugation (2000 *g*, 10 min, RT).

### 2.5 Preparation of UV light–killed *E. coli*

An antibiotic-sensitive strain of *E. coli* (NCTC 10418) from the UK Health Security Agency (formerly Public Health England) was cultured on Luria Bertani (LB) agar plates overnight at 37 °C. A single colony-forming unit was transferred to a 10 mL LB broth starter culture, incubated for 6 h at 37 °C, and 750μL transferred to 750 mL LB broth for overnight incubation (220 rpm, 16 h, 37 °C). The next morning, the broth was centrifuged (4000 *g*, 20 min, 4 °C), washed twice in PBS (4000 *g*, 20 min, 4 °C), and sterilized for 2 h with a 302 nm UV light transilluminator (UVP). The UV-killed *E. coli* were then washed twice in sterile PBS (4000 *g*, 20 min, 4 °C) and sterility was confirmed by the University College London Microbiology Laboratory.

To quantify UV-killed *E. coli* at a wavelength of light absorbed by *E. coli* but not by MB, an optical density (OD) growth curve was generated using 420 nm light. Aliquots of 1 mL from the above 750 mL culture broth were sampled and analyzed every 30 min. At each time point, OD at 420 nm (OD_420_) was measured, and colony-forming units were counted by serial dilution, overnight culture, colony counting, and dilution factor multiplication.

### 2.6 Labeling *E. coli* with MB

A total of 5 × 10^8^ UV-killed *E. coli* in 2 mL PBS were centrifuged (6000 *g*, 10 min, RT) in 2 mL round-bottom Eppendorfs (Appleton Woods), and the supernatant was removed. At a 60° angle, pellets were labeled directly with 1μL 1% MB overnight at RT, resuspended in 2 mL PBS, and centrifuged (6000 *g*, 2 min, RT), and the washing solution was removed. Where stated, 0.5% or 0.3% MB was used instead of 1% MB. MBEC pellets were resuspended in 200μL sterile 0.9% sodium chloride (NaCl) and quantified by OD_420_. The quantity of MB attached to MBEC was calculated by increasing the concentration of pelleted *E. coli*, labeling with 1.3μL MB, and measuring the fall in MB concentration remaining in the MBEC washing solutions using the following formula: mass = concentration × volume.

To assess the temporal stability of the MB label, MBEC was incubated at 37 °C for 3 h prior to flow cytometry. To confirm that their MB fluorescence originated from the labeled *E. coli* and not from their supernatants, the MB concentration of the labeling, washing, and final resuspension solutions were measured by spectrophotometry. For the same reason, amine nonreactive compensation beads (Invitrogen), which do not absorb MB, were resuspended in the final MBEC supernatants before flow cytometry.

### 2.7 Leukocyte coculture with MBEC

As part of a phagocytosis assay, neutrophils, monocytes, monocyte-derived macrophages, or all leukocytes were suspended in RPMI + 10% autologous serum and cocultured for 60 min (unless stated otherwise) with either MBEC or unlabeled *E. coli* at the multiplicity of infection (MOI) provided. In all experiments, gated SSC^hi^FSC^hi^ granulocytes (of which ∼95% are neutrophils) served as the unstained and single-stained controls for flow cytometry.^31^ The flow cytometry gates separating MB^lo^ from MB^hi^ neutrophils (Supplementary Data 1) were defined using neutrophils cocultured with unlabeled *E. coli* (i.e. a fluorescence-minus-one control for MB). In some experiments, the previous phagocytosis assay was modified to answer different experimental questions. Therefore, where stated, neutrophils were (1) pretreated with 10μM cytochalasin B in dimethyl sulfoxide for 2 h, (2) supplemented with MB dye at the stated concentrations, or (3) after MBEC coculture resuspended in 100μL fixation-permeabilization buffer (eBioscience).^32^ To test the effect of MB on phagocytosis, pHrodo green *E. coli* BioParticles (Invitrogen) prepared in RPMI + 10% fetal calf serum according to the manufacturer's recommendations served as an alternative fluorescently labeled particle to MBEC for measuring phagocytosis ex vivo (MOI = 20).

### 2.8 Flow cytometry

Before flow cytometry, leukocytes from the above assays were centrifuged (500 *g*, 5 min, RT), resuspended in 50μL of Brilliant Stain Buffer (BD) and 50μL of antibody solution (30 min, 4 °C), washed in FACS buffer (500 *g*, 5 min, RT), and fixed in 2% PFA (Thermo Fisher Scientific). For conventional flow cytometry, the antibody solution contained FACS buffer and Live/Dead Zombie UV (BioLegend; 1:100), CD45 BV785 (BioLegend; 1:100), CD3 PE dazzle (BioLegend; 1:100), CD19 BV605 (BioLegend; 1:100), CD56 BV605 (BioLegend; 1:100), CD4 PE-Cy7 (BioLegend; 1:100), CD8 BV510 (BioLegend; 1:100), HLA-DR BV421 (BioLegend; 1:50), CD66b FITC (BioLegend; 1:50), Siglec8 PE (BioLegend; 1:50), CD14 BUV805 (BD; 1:100), CD16 BUV395 (BD; 1:100), CD62L BUV737 (BD; 1:100), and CD45RA BV711 (BioLegend; 1:100). For spectral flow cytometry, the antibody solution contained FACS buffer and Live/Dead Zombie UV (BioLegend; 1:200), CD45 SparkViolet538 (BioLegend; 1:100), CD3 PE (BioLegend; 1:100), CD19 PE (BioLegend; 1:100), HLA-DR PE (BioLegend; 1:100), CD56 PE (BioLegend; 1:100), CD66b Pacific Blue (BioLegend; 1:100), Siglec8 BUV395 (BD; 1:200), CD11a FITC (BioLegend; 1:200), CD11b SparkBlue 550 (BioLegend; 1:200), CD14 SparkBlue 574 (BioLegend; 1:200), CD16 BV570 (BioLegend; 1:200), CD33 BUV496 (BD; 1:200), CD11c BV480 (BD; 1:200), CCR7 SparkYellowGreen 581 (BioLegend; 1:200), CD15 BUV563 (BD; 1:200), CD62L BV421 (BioLegend; 1:200), CXCR2 PE-Dazzle594 (BioLegend; 1:200), and CXCR4 BV605 (BioLegend; 1:200). The gating strategy for each panel is shown in Supplementary data 1.

### 2.9 Fluorescence microscopy

For fluorescence microscopy, single-cell leukocyte suspensions were centrifuged (800 rpm, 5 min, RT; Shandon Cytospin 2) onto Polysine-coated microscope slides (Thermo Fisher Scientific), fixed in 2% PFA for 10 min, and washed twice in PBS. After 60 min protein blockade in BlockAid solution (Life Technologies) leukocytes were stained with FITC-labeled anti-CD3, anti-CD14, anti-CD19, or anti-CD66b antibodies (BioLegend) for 60 min at RT (1:100). Leukocytes were washed 3 times in PBS and mounted in ProLong Glass Antifade Mountant (Invitrogen) with or without Hoechst stain, before image acquisition at 63 × magnification using oil-immersion fluorescence microscopy (Leica TCS SP8).

For live imaging, 2.5 × 10^4^ neutrophils were transferred to a chamber slide (Ibidi; Thistle Scientific) containing 10% autologous serum. Image acquisition was performed every 6 s with a Zeiss LSM980 Airyscan live imaging fluorescence microscope, 63 × oil-immersion lens, and differential interference contrast HSII Wollaston prism. Unlabeled *E. coli* or MBEC were added shortly after starting image acquisition (MOI = 100), which continued for 20 min.

### 2.10 Skin model of acute inflammation

A 5 cm^2^ area of skin 7 cm below the antecubital fossae on both volar forearms of healthy volunteers was shaved, marked, and cleaned (Universal Alcotip). A total of 6 × 10^7^ UV-killed *E. coli* or MBEC were prepared in sterile 0.9% NaCl as described previously and injected intradermally with a 1 mL syringe (BD) and sterile 30G needle (BD). Dermal blood flow was measured by laser doppler imaging (Moor Instruments), and a skin biopsy or suction blister was performed at the prespecified time points as described subsequently.

For skin biopsies, the site was cleaned with 2% chlorhexidine and anaesthetized with 2% lignocaine (Hameln pharma). A 5 mm punch biopsy (Stiefel) was performed, cryopreserved in Optimum Cutting Temperature compound (VWR Chemicals), cryosectioned at 50μM (HM525 NX CryoStat), and transferred to Polysine-coated microscope slides. Protein blockade, anti-CD66b staining, mounting, and image acquisition were performed as described previously. The biopsy site was then cleaned, sutured, and covered, before the sutures were removed after 14 d.

For suction blisters, the host laboratory's technique was used, as previously described.^20^ Briefly, a sealed cup with a 10 mm aperture was connected to a negative pressure instrument (Electronic Diversities NP-4) and secured over the site of intradermal MBEC or *E. coli* injection. Suction was increased until a single epidermal blister filled the aperture. The negative pressure was then reduced, and the cup was removed. The blister roof was pierced with a sterile 18G needle (BD) and the exudate transferred to a 96-well plate (Thermo Fisher Scientific) prefilled with 50μL ice-cold 0.5μM EDTA in PBS. The blister was then deroofed, cleaned with 0.5% cetrimide (Boots), and covered with a sterile dressing (Mepore). The inflammatory exudate was centrifuged (1000 *g*, 5 min, 4 °C) and the blister leukocytes were stained with conjugated antibodies as described previously, and analysed by flow cytometry. To measure blister fluid endotoxin levels (i.e. bacterial clearance), the cell-free exudate was diluted (1:50) in endotoxin-free LAL reagent water and analyzed using the Endosafe NexGen portable endotoxin testing system (Charles River).

### 2.11 Data analysis and statistical calculations

At the default detector voltages (unless otherwise stated), conventional flow cytometry was performed using a BD LSR Fortessa X20 and spectral flow cytometry using a Sony ID7000 Spectral Cell Analyzer. For 2-dimensional flow cytometry analysis, flow cytometry standard files were exported to FlowJo (TreeStar) and numerical data to GraphPad Prism (GraphPad Software). For multidimensional flow cytometry analysis, CD45^hi^Lin^lo^Siglec8^lo^CD66b^hi^ neutrophils or CD45^hi^Siglec8^lo^ leukocytes (i.e. excluding Siglec8^hi^ eosinophils) were imported to *R* and analyzed using the referenced CyTOF workflow.^33^

For statistical comparison, the means between 2 groups were compared with Student's *t* tests and the means between 3 or more groups with 1 independent variable using 1-way analysis of variance and Dunnett's multiple comparisons test. The means between 3 or more groups with 2 independent categorical variables were compared with 2-way analysis of variance and Dunnett's multiple comparisons test. Statistical significance was shown as follows: *P ≤ 0.05, **P ≤ 0.01, ***P ≤ 0.001, ****P ≤ 0.0001, and ns (not significant).

## 3. Results

### 3.1 MB can be detected by fluorescence microscopy and flow cytometry

**Fig. 2.**
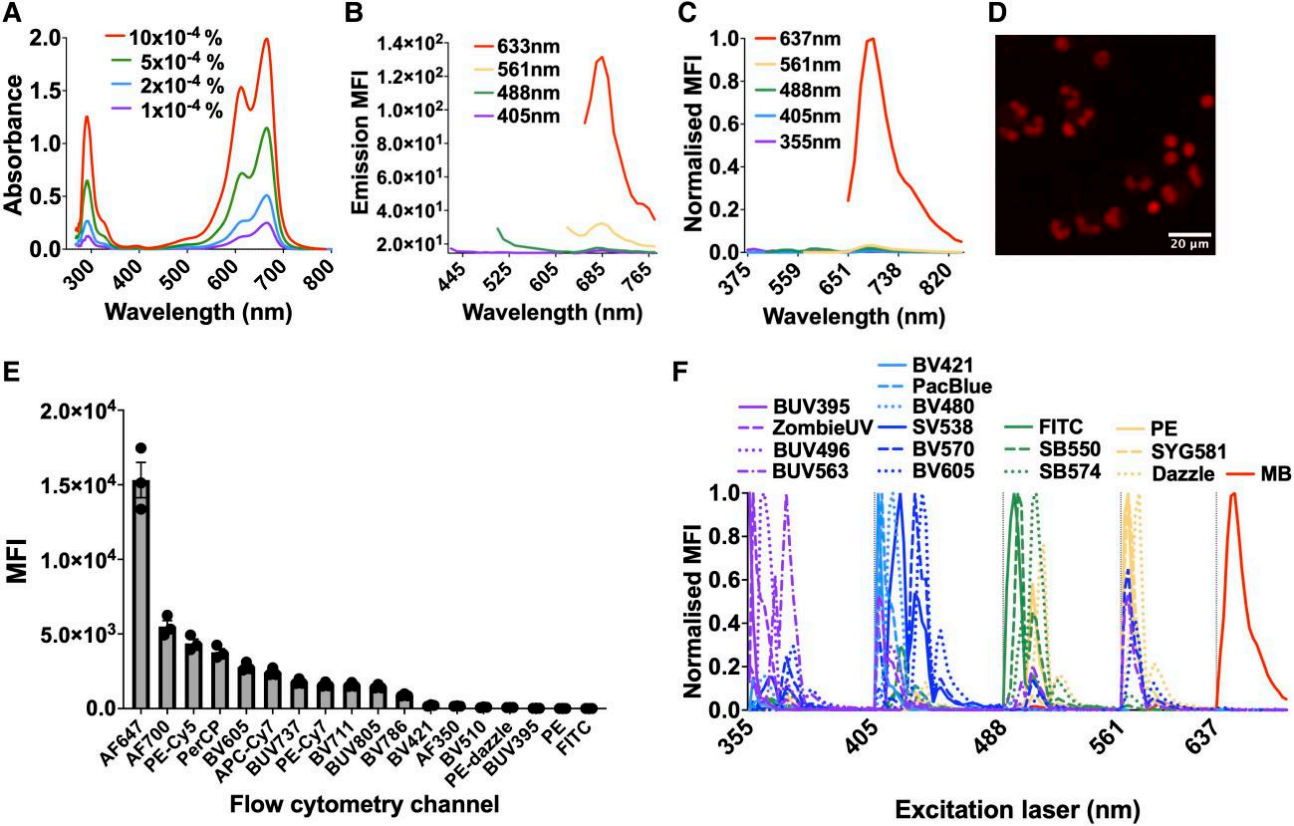
Optimizing MB detection by flow cytometry and fluorescence microscopy. (A) Absorbance spectra of 4 concentrations of MB detected by spectrophotometry. (B) Emission spectra of 1% MB after excitation by 4 fluorescence microscopy lasers using the Leica TCS SP8 HyD detector. (C) Emission spectra of MB-labeled leukocytes after excitation by 5 spectral flow cytometry lasers, with the 637 nm detector set sensitivity increased to 70 V and all values normalized so that the peak equals 1. (D) Representative cytospin of MB-labeled leukocytes, as detected by fluorescence microscopy after red laser excitation. (E) MFI of MB-labeled leukocytes in all conventional flow cytometry channels at the default detector voltages, in rank order of MFI (n = 3). (F) Ribbon plot showing emission spectrum of MB alongside 16 conjugated antibody fluorophores, after excitation by all 5 spectral flow cytometry lasers. BV = brilliant violet; BUV = brilliant ultraviolet; PacBlue = pacific blue; PE = phycoerythrin; SB = spark blue; SV = spark violet; SYG = spark yellow green.

To begin developing a new in vivo phagocytosis model using the intradermal injection of MBEC, we first confirmed the excitation and emission wavelengths of MB. MB was maximally absorbent at 665 nm (Fig. 2A) and hence maximally excited by the red laser (Fig. 2B). This was assessed using spectral flow cytometry (Fig. 2C) and fluorescence microscopy (Fig. 2D) by analyzing the emission of fixed MB-labeled leukocytes after red laser excitation. Fixed leukocytes were used because in live cells MB is reduced to its transparent form leucomethylene blue, explaining its traditional use as a viability stain. At the default flow cytometry voltages, the best conventional flow cytometry channel to detect MB was the AF647 channel (Fig. 2E), which combines red laser excitation with a 655 to 685 nm detector set. Reserving the red laser exclusively for MB detection allowed simultaneous detection of 13 conjugated antibodies during conventional flow cytometry (see compensation matrix in section 5.4), and 16 conjugated antibodies during spectral flow cytometry (Fig. 2F), in turn allowing multidimensional analysis of leukocyte surface marker expression alongside MB detection (Supplementary Data 1). Hence, alongside leukocyte surface molecule expression, MB could be detected after red laser excitation by flow cytometry and fluorescence microscopy.

### 3.2 *E. coli* can be labeled with MB

**Fig. 3.**
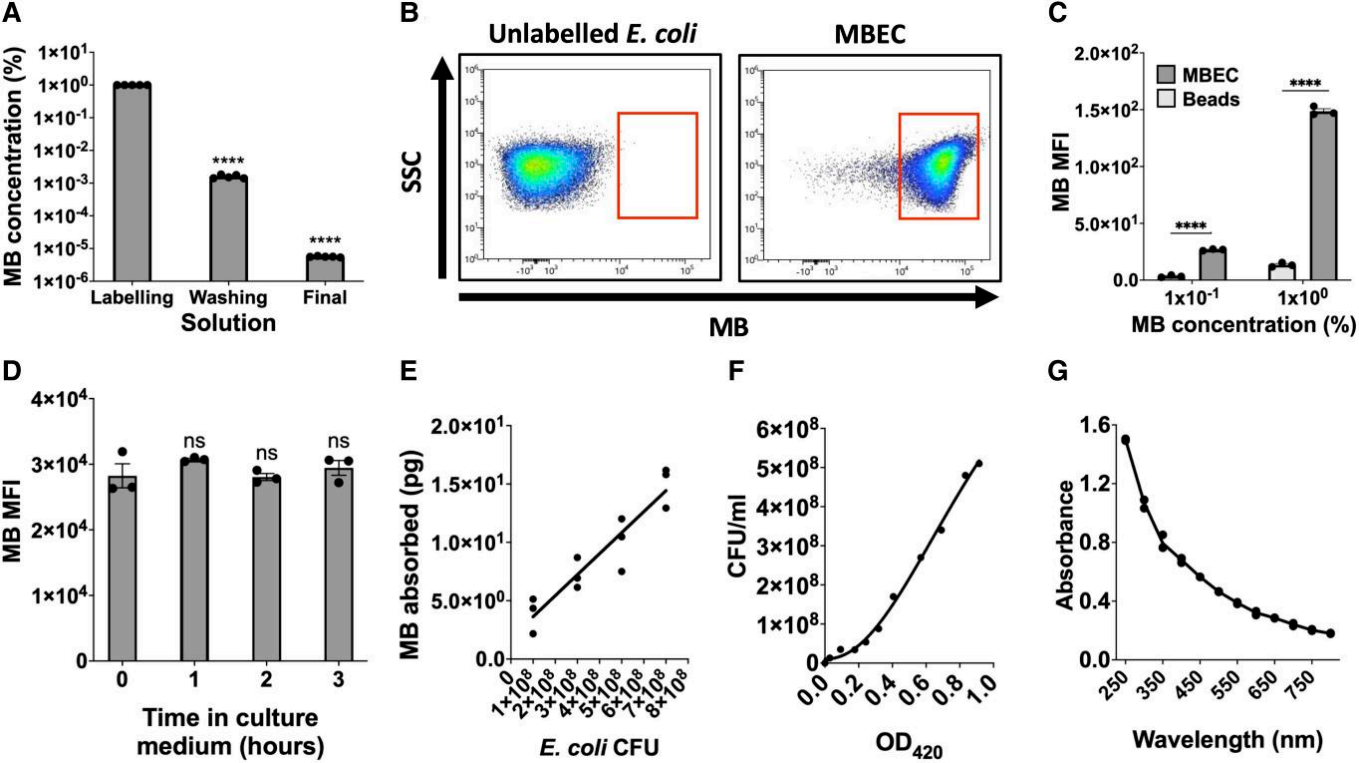
Labeling, washing, and quantifying MBEC. (A) Fall in MB concentration between the *E. coli* labeling, washing, and final resuspension solutions during the process of labeling UV-killed *E. coli* with MB to produce MBEC (n = 5). (B) Representative spectral flow cytometry plots of unlabeled *E. coli* (left) and MBEC (right), showing side scatter (SSC) (y-axis) and MB fluorescence values (x-axis). (C) MB MFI of MBEC compared with MB-inert compensation beads suspended in the same supernatants (n = 3). (D) MB MFI of MBEC during a 3-h incubation in RPMI + 10% fetal calf serum at 37 °C (n = 3). (E) Increasing quantities (i.e. colony-forming units [CFUs]) of *E. coli* were labeled with 1.3μL of 1% MB, and the absorption of MB by the *E. coli* was inferred from the concentration of MB remaining in the washing solutions after MB-labeling and resuspension in PBS, using the following formula: mass = concentration × volume (n = 3). (F) The OD growth curve of unlabeled *E. coli* using 420 nm light. (G) Absorbance spectrum of unlabeled *E. coli* (n = 3). Significance reported using a paired Student's *t* test (C) or 1-way analysis of variance with Dunnett's multiple comparisons test (A, D). ****P ≤ 0.0001. ns = not significant.

To measure phagocytosis ex vivo, we first determined whether *E. coli* could be labeled with MB. Unlabeled antibiotic-sensitive *E. coli* were cultured overnight, killed with UV light, labeled with 1% MB, and washed in PBS. The concentration of MB in MBEC supernatants decreased from 1% during labeling to 1 × 10^−5^% after washing (Fig. 3A). Furthermore, when analyzed by flow cytometry, labeled MBEC had significantly higher MB fluorescence than unlabeled *E. coli* (Fig. 3B).

To confirm that this MB fluorescence originated from the MBEC and not from any residual MB remaining in their supernatants after washing, MBEC geometric MFI was compared with amine nonreactive beads suspended in the same MBEC supernatants. These amine nonreactive beads are similar in size to *E. coli* but do not bind organic dyes such as MB. MBEC had significantly higher MB fluorescence than the beads, suggesting that the MB fluorescence in the MBEC originated from the labeled bacteria and not from their supernatants (Fig. 3C). Next, MBEC were incubated in culture medium for 3 h. During this time, they did not lose their MB label (Fig. 3D), showing that the MB labeling of UV-killed *E. coli* was stable over time.

To calculate the amount of MB attached to *E. coli* and therefore whether the MB label could affect immune responses, the quantity of MB attached to MBEC was inferred from the concentration of MB remaining in the washing solution after labeling increasing quantities of UV-killed *E. coli* with the same volume of 1% MB. The amount of MB bound to the labeled *E. coli* was in the order of picograms (Fig. 3E), 11 orders of magnitude below the intravenous dose of MB used in clinical practice (1 to 2 mg/kg) for the treatment of conditions such as acquired methaemaglobinaemia.^34, 35^

Before intradermal MBEC injection, accurate bacterial quantification was also important. However, UV-killed *E. coli* are usually quantified using spectrophotometry at OD_600_, a wavelength of light absorbed by MB (Fig. 2A).^36^ Therefore, to infer MBEC concentration independent of MB labeling, a new standard curve (Fig. 3F) was generated using OD_420_, a wavelength of light absorbed by *E. coli* (Fig. 3G) but not by MB (Fig. 2A). Hence, UV-killed *E. coli* could be labeled with MB, washed, and accurately quantified.

### 3.3 MBEC can be used to measure phagocytosis ex vivo

**Fig. 4.**
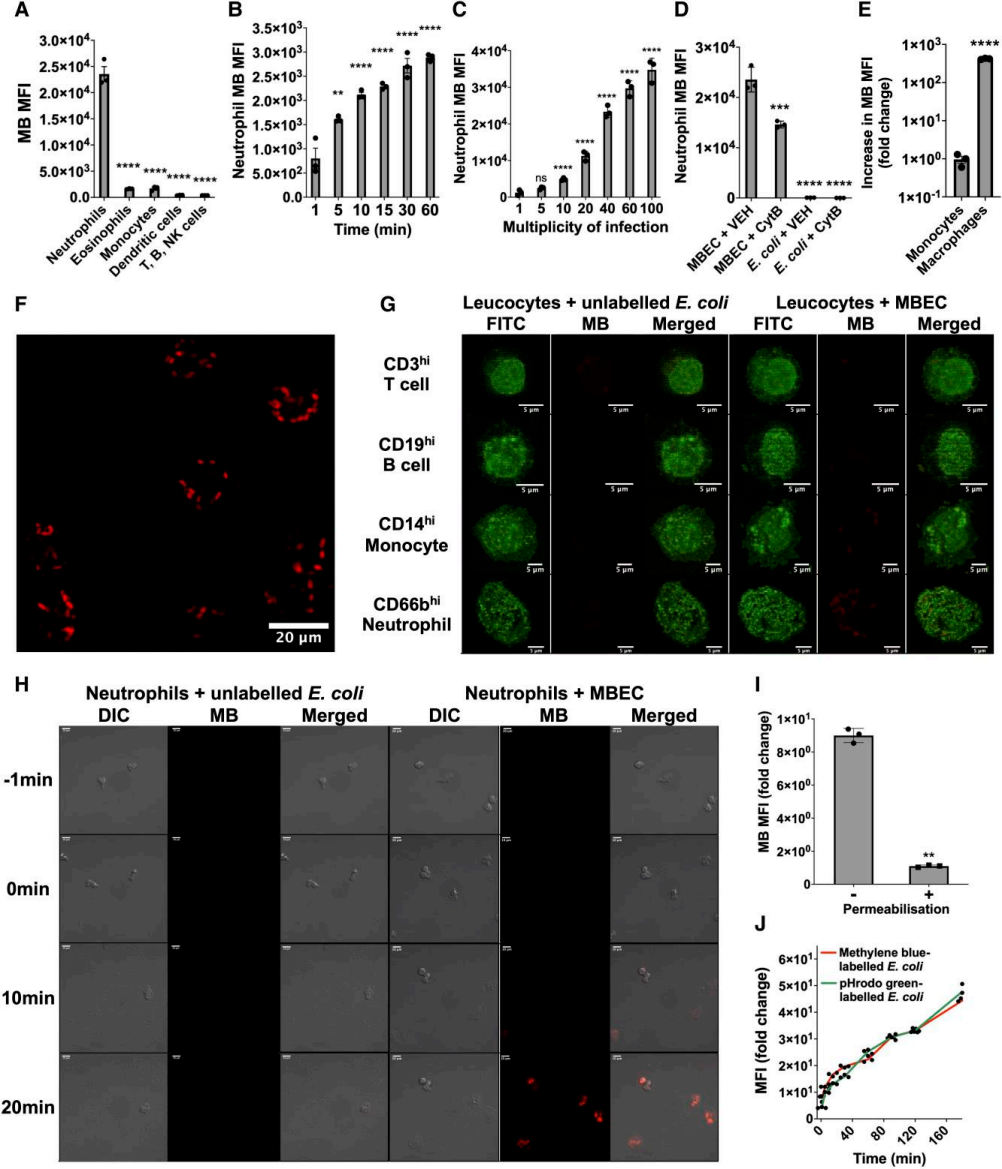
Ex vivo coculture of MBEC with human circulating leukocytes. (A) MB MFI of circulating leukocyte subsets after 60 min MBEC coculture (n = 3, MOI = 30). (B) MB MFI of isolated neutrophils at different time points during 60 min MBEC coculture (n = 3, MOI = 10). (C) MB MFI of isolated neutrophils after 60 min MBEC coculture at increasing MOI (n = 3). (D) MB MFI of isolated neutrophils after 60 min coculture with MBEC or unlabeled *E. coli*, with or without pretreatment with 10μM cytochalasin B to inhibit phagocytosis (n = 3, MOI = 30).^32^ (E) Fold change MB MFI during MBEC coculture with monocyte-derived macrophages or naïve circulating monocytes, calculated as fold change from control cells without bacteria (n = 3, MOI = 100). (F) Fluorescence microscopy of circulating leukocytes after 60 min MBEC coculture (MOI = 40, Leica TCS SP8). (G) Fluorescence microscopy of cellular distribution of MB^hi^ cellular inclusions in 4 leukocyte subsets after 60 min coculture of circulating leukocytes with unlabeled *E. coli* (left) or MBEC (right, MOI = 30). (H) Still frames from a representative live imaging video (Supplementary Data 2) of isolated neutrophils cocultured with unlabeled *E. coli* (left) or MBEC (right) for 20 min during real-time differential interference contrast (DIC) and fluorescence imaging microscopy (MOI = 100; Zeiss LSM980 Airyscan). (I) Effect of cell membrane permeabilization buffer treatment on neutrophil MB MFI after 60 min MBEC coculture (n = 3, MOI = 30), calculated as the fold change from control neutrophils without MBEC. (J) MFI fold-change of pHrodo green or MB during coculture of neutrophils from healthy volunteers with pHrodo green *E. coli* BioParticles or MBEC, respectively (n = 3, MOI = 20), calculated as the fold change from control neutrophils without bacteria. Significance reported using a paired Student's *t* test (H) or 1-way analysis of variance with Dunnett's multiple comparisons test (A–E). **P ≤ 0.01, ***P ≤ 0.001, ****P ≤ 0.0001. CytB = cytochalasin B; ns = not significant; VEH = vehicle.

To understand whether MBEC could be used to measure bacterial phagocytosis ex vivo, circulating leukocytes from healthy volunteers were cocultured with MBEC or unlabeled *E. coli*, stained with conjugated antibodies, and analyzed by flow cytometry. Coculture of leukocytes with MBEC caused MB to transfer primarily into neutrophils (Fig. 4A). The absence of MB in the other less phagocytic leukocyte subsets suggested that this transfer was caused by phagocytosis, and not by diffusion of any residual MB remaining in the MBEC supernatants. This was supported when the MB fluorescence of neutrophils during incubation with MBEC increased over time (Fig. 4B) and with increasing MOI (Fig. 4C) but not with unlabeled *E. coli* controls (Fig. 4D).

This was further supported when (1) cytochalasin B pretreatment (which inhibits phagocytosis) reduced MB transfer into neutrophils (Fig. 4D), (2) MB uptake was higher in monocyte-derived macrophages than naïve circulating monocytes (Fig. 4E), and (3) the leukocyte staining pattern after MBEC coculture (Fig. 4F) was different to after direct labeling of fixed leukocytes with MB dye (Fig. 2D).^32^ Specifically, the leukocytes cocultured with MBEC contained MB^hi^ inclusions resembling phagolysosomal staining, but direct labeling of fixed leukocytes with MB produced nuclear staining, suggesting that staining during MBEC coculture was caused by phagocytosis and not by the diffusion of MB into leukocytes from MBEC supernatants.^37–41^ Staining leukocytes after MBEC coculture with conjugated antibodies confirmed that these MB^hi^ inclusions (Fig. 4F) were absent after coculture with unlabeled *E. coli*, primarily observed in neutrophils, and present to a lesser extent in naïve circulating monocytes (Fig. 4G).

The hypothesis that MBEC phagocytosis caused these MB^hi^ inclusions was confirmed by the live microscopy imaging videos in Supplementary Data 2 and still image frames from these videos in Fig. 4H. During MBEC coculture, neutrophils internalized MBEC, forming intracellular and non-nuclear MB^hi^ inclusions in keeping with phagolysosomal staining (Supplementary Data 2B).^37–41^ Again, these inclusions were different from direct staining of fixed leukocytes with MB (Fig. 2D) and absent during coculture with unlabeled *E. coli* (Supplementary Data 2A and Fig. 4H).

When neutrophils were treated with membrane permeabilization buffer after coculture with MBEC, MB fluorescence was significantly reduced (Fig. 3I). This was likely due to dissipation of MB through perforated phagolysosomal and extracellular membranes, further supporting the value of MBEC in measuring bacterial phagocytosis. Finally, when neutrophils were cocultured with either MBEC or pH-sensitive pHrodo green *E. coli* BioParticles at an equivalent MOI (20), both MB and pHrodo green dye accumulated in neutrophils at an equivalent rate (Fig. 4J).

Together, these data suggested that MBEC were equally effective to a commercial fluorescent probe in measuring phagocytosis ex vivo, while also being safe for human injection.

### 3.4 MBEC can also measure phagolysosomal oxidation ex vivo

**Fig. 5.**
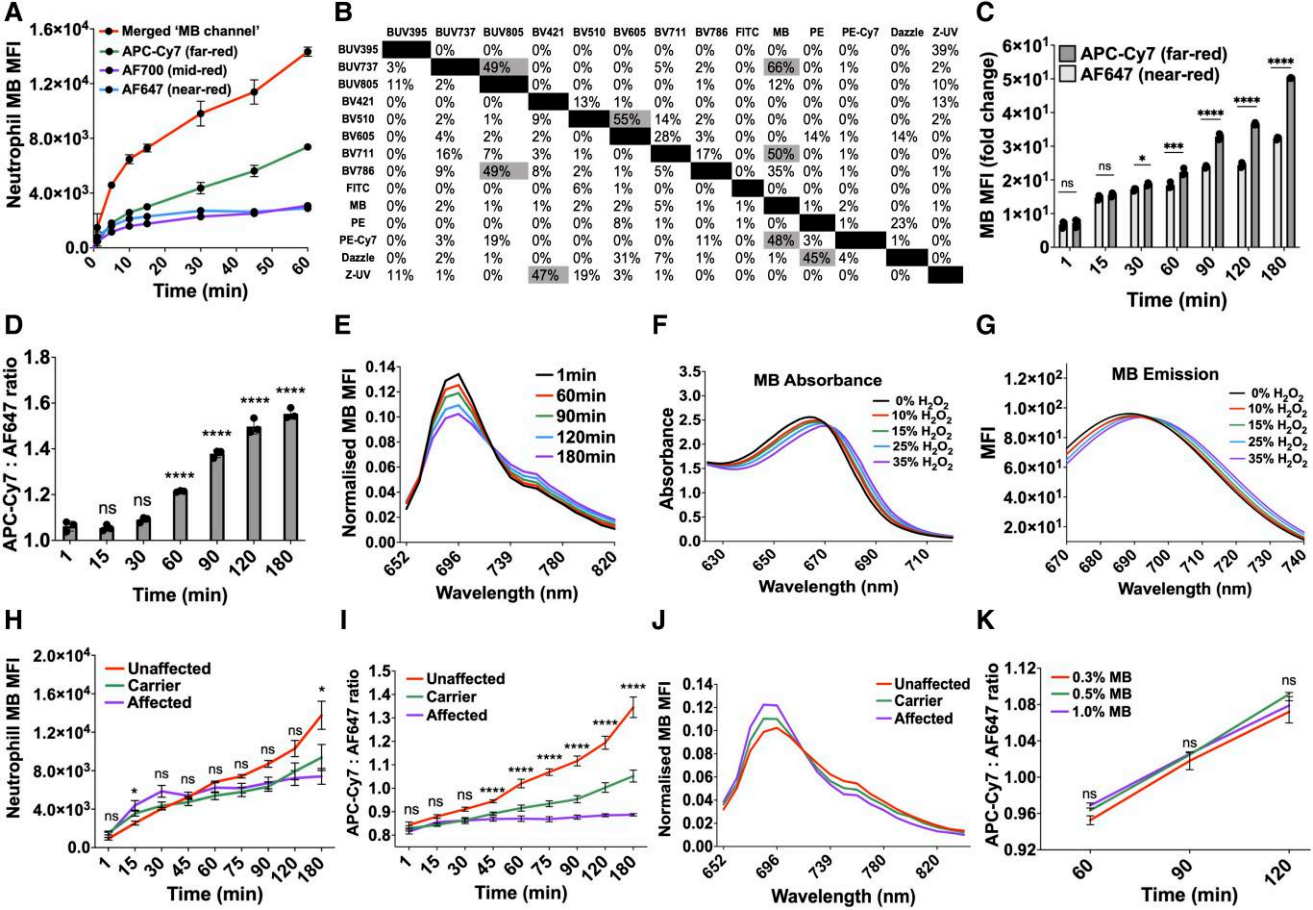
Red shift in MB fluorescence during ex vivo MBEC phagocytosis. (A) Neutrophil MB MFI in the 3 red-excited red-emitting conventional flow cytometry channels (AF647, AF700, and APC-Cy7) at above-default detector voltages (AF647 = 650, AF700 = 650, APC-Cy7 = 600) during ex vivo MBEC coculture (MOI = 10). This included the merged “MB channel”, in which data from all 3 channels were combined post hoc using FCS Express software (n = 3). (B) Representative conventional flow cytometry compensation matrix when MB was detected alongside 13 conjugated antibody fluorophores (Supplementary Data 1, overlap values of >40% highlighted). (C) Comparison of neutrophil MB MFI using the AF647 (near-red) and APC-Cy7 (far-red) channels during ex vivo MBEC coculture (n = 3). (D) Data in panel C are presented as the far-to-near-red (i.e. APC-Cy7:AF647) channel ratio. (E) Neutrophil MB emission spectra during ex vivo MBEC coculture analyzed by spectral flow cytometry and corrected for total MB MFI at each time point by dividing each data point by the sum of all data points for each time point, to allow for side-by-side comparison of MB emission spectra over time (MOI = 20). (F, G) Absorbance (F) and emission (G) spectra of MB when combined in vitro with increasing concentrations of hydrogen peroxide (H_2_O_2_). (H, I) Effect of gp91phox chronic granulomatous disease status on neutrophil MB MFI (H) and neutrophil APC-Cy7:AF647 channel ratio (I) during ex vivo neutrophil MBEC coculture (n = 4). (J) Data from the 180 min time point in panel I analyzed using spectral flow cytometry. (K) Effect of *E. coli* MB labeling concentration on neutrophil MB red shift (APC-Cy7:AF647 ratio) during ex vivo neutrophil MBEC coculture (n = 3). Significance reported using a paired Student's *t* test (C), 1-way analysis of variance with Dunnett's multiple comparisons test (D), or 2-way analysis of variance with Tukey's multiple comparisons test (comparing unaffected vs affected [H, I] and comparing 0.3% with 1% MB [K]). *P ≤ 0.05, ***P ≤ 0.001, ****P ≤ 0.0001. ns = not significant.

Because the red emission spectrum of MB (Fig. 2B) was wider than the detection range of the AF647 flow cytometry channel (655 to 685 nm), using the AF647 channel alone to detect MB excluded the red-emitting MB fluorescence outside this range, reducing the sensitivity of conventional flow cytometry to MB. To address this, fluorescence data from the AF647 channel (655 to 685 nm) were combined with the AF700 (705 to 750 nm) and APC-Cy7 channels (750 to 810 nm) post hoc using FCS Express software, a novel concept not previously described in the literature. This created a merged, virtual “MB channel”, with the wider detection range 655 to 810 nm, increasing the amount of gathered light and therefore sensitivity of conventional flow cytometry to MB (benefitted because cellular autofluorescence is low at these wavelengths). This was supported by the MFI data in Fig. 5A, which shows the AF647, AF700, APC-Cy7, and merged “MB channel” MFIs for neutrophils during 60 min MBEC coculture. Despite this wider detection range, combining detection of neutrophil MB (i.e. phagocytosed MBEC) with 13 conjugated antibody fluorophores still produced satisfactory overlap values during conventional flow cytometry fluorophore compensation (Fig. 5B).

During analysis of these 3 red emission–detecting conventional flow cytometry channels, we noticed that ex vivo MBEC phagocytosis caused the emission spectrum of MB in neutrophils to “shift” from the near-red AF647 channel toward the far-red APC-Cy7 channel (Fig. 5A, C). Hereafter referred to as “red shift,” this change in MB emission from the near-red to the far-red side of the light spectrum was quantified by dividing the far-red APC-Cy7 channel MFI by the near-red AF647 MFI to generate an APC-Cy7:AF647 ratio (Fig. 5D). This red shift was confirmed using spectral flow cytometry to be caused by a fall in the 696 nm primary emission peak and an increase in the 760 nm shoulder emission peak during MBEC phagocytosis (Fig. 5E).

We next investigated the cause of this red shift in MB emission during MBEC phagocytosis, with the hypothesis that it could be caused by phagolysosomal oxidation, an important bactericidal process in neutrophils.^42^ To test this, we combined MB with increasing concentrations of hydrogen peroxide (H_2_O_2_) in vitro and observed a noticeable red shift in MB fluorescence (Fig. 5F, G).

To assess whether this red shift was caused by phagolysosomal oxidation ex vivo, MBEC was cocultured with neutrophils from patients with X-linked (gp91phox) CGD, who due to defective nicotinamide adenine dinucleotide phosphate oxidase activity cannot effectively oxidize their phagolysosomes.^43^ Despite similar phagocytic capacity between neutrophils from CGD patients and healthy control subjects (Fig. 5H), the red shift in MB fluorescence during MBEC phagocytosis was absent in CGD patients, with female carriers manifesting an intermediate phenotype consistent with lyonization of the abnormal X chromosome in approximately 50% of neutrophils (Fig. 5I, J). This suggested that the red shift in neutrophil MB emission observed during MBEC phagocytosis was caused by phagolysosomal oxidation and not by MB accumulation. This was confirmed when the red shift in MB fluorescence persisted despite reducing the concentration of MB used to label the UV-killed *E. coli* prior to neutrophil MBEC coculture from 1% to 0.5% and 0.3% (Fig. 5K). Together, these data showed that in addition to bacterial phagocytosis, MBEC can also be used to independently measure phagolysosomal oxidation in humans ex vivo.

### 3.5 Inflammation caused by MBEC is similar to that caused by unlabeled *E. coli*

**Fig. 6.**
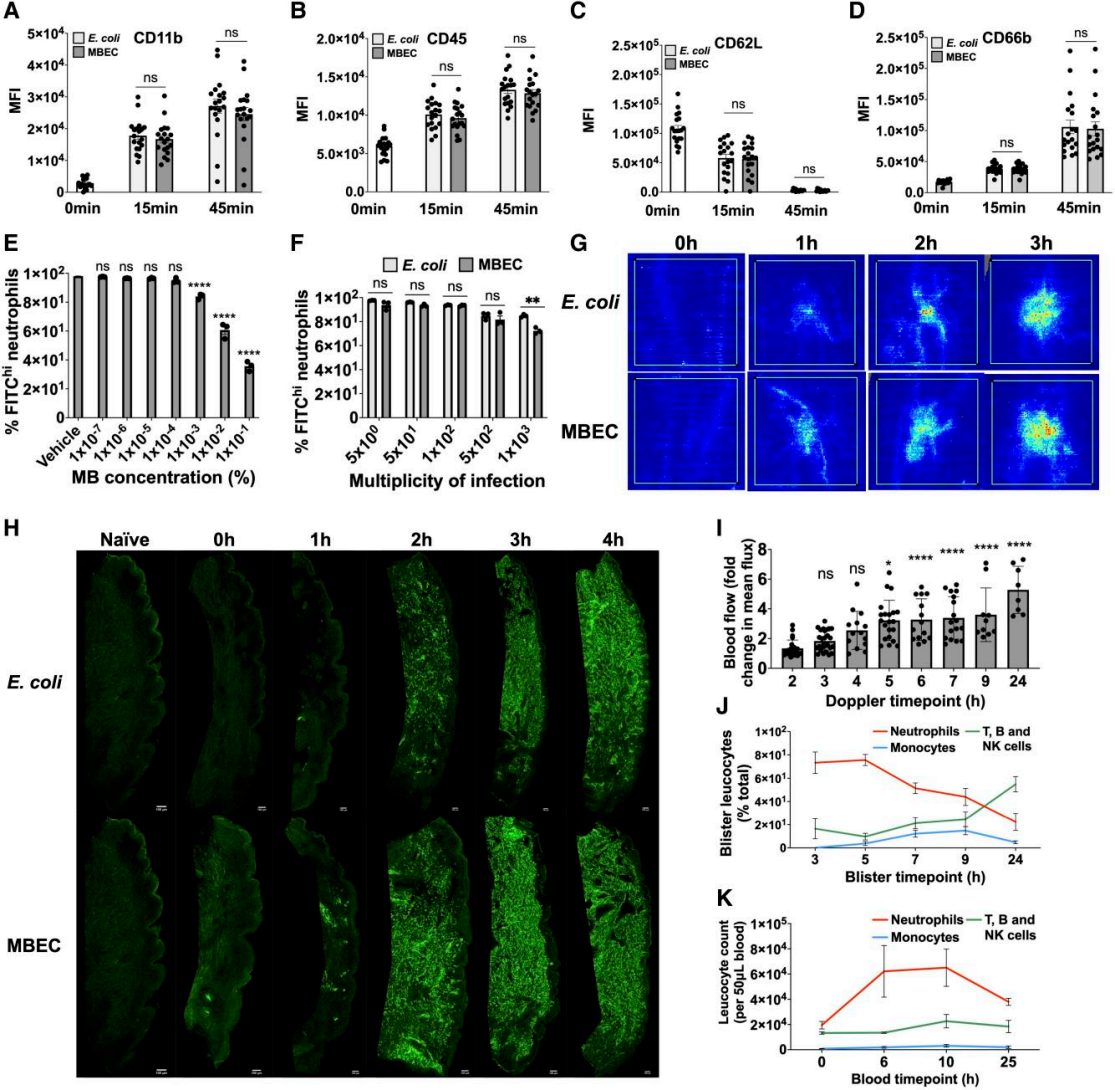
Effect of MB on the immune response in humans. (A–D) Surface membrane expression of CD11b (A), CD45 (B), CD62L (C), and CD66b (D) on neutrophils during ex vivo coculture with unlabeled *E. coli* or MBEC (n = 19, MOI = 5) in RPMI + 10% FCS. (E, F) Effect of MB (E) or MBEC (F) concentration on ex vivo neutrophil phagocytosis of pHrodo green *E. coli* BioParticles (n = 3). (G) Comparison of dermal vascular hyperemia between intradermal unlabeled *E. coli* or MBEC injections in healthy volunteers, measured by laser Doppler blood flow imaging. (H) Representative skin biopsy sections comparing dermal CD66b^hi^ neutrophil infiltration over time after intradermal unlabeled *E. coli* or MBEC injections in healthy volunteers imaged using fluorescence microscopy (see Supplementary Data 3B for Hoechst counterstaining). (I–K) Effect of intradermal MBEC injection on dermal vascular hyperemia (I), relative dermal leukocyte abundance (J), and absolute circulating leukocyte abundance (K) in healthy volunteers (n = 6) (see Supplementary Data 3C for absolute blister leukocyte abundances). Significance reported using paired Student's *t* tests (A–D, F) or 1-way analyses of variance with Dunnett's multiple comparisons test (E, I). *P ≤ 0.05, **P ≤ 0.01, ****P ≤ 0.0001. NK = natural killer; ns = not significant.

Having demonstrated that MBEC can be used to measure phagocytosis and phagolysosomal oxidation ex vivo, we next compared the immune response between MBEC and unlabeled *E. coli*. This was to understand whether MBEC were equally inflammatory to the unlabeled *E. coli* used in the existing skin challenge model, and whether MB labeling had any immunomodulatory effect on the pattern of acute inflammation in vivo.^20^ Neutrophils cocultured with MBEC ex vivo showed equivalent changes in activation status to those cocultured with unlabeled *E. coli*, whereby CD11b, CD45, and CD66b were upregulated (Fig. 6A, B and D, respectively) and CD62L was shed (Fig. 6C).

The functional effect of MB on bacterial phagocytosis was then tested using pHrodo green *E. coli* BioParticles, which are detected in the FITC channel and therefore do not spectrally overlap with MB (Figs. 2E and 5B). MB dissolved in the phagocytosis assay culture medium inhibited pHrodo green *E. coli* BioParticle phagocytosis by neutrophils at MB concentrations above 1 × 10^−3^% (Fig. 6E); however, this was 2 orders of magnitude over the MB concentration present in MBEC supernatants (Fig. 3A).

To replicate the MBEC model more closely, the effect of MB as an *E. coli* label on phagocytosis was then tested by measuring phagocytosis of pHrodo green *E. coli* BioParticles in combination with increasing concentrations of either MBEC or unlabeled *E. coli*. When used as an *E. coli* label, MB inhibited phagocytosis at an MOI of over 500, 3 orders of magnitude above the MOI present in human skin after intradermal *E. coli* injection (Fig. 6F).^44^

Having demonstrated that at the concentrations present in the MBEC model MB had little effect on neutrophil function ex vivo, the effect of MB on acute inflammation was investigated in humans in vivo. MBEC and unlabeled *E. coli* were injected intradermally into the left and right volar forearms of healthy volunteers, respectively. Laser Doppler imaging showed that the increase in blood flow after intradermal MBEC injection was similar to after unlabeled *E. coli* injection (Fig. 6G). Dermal neutrophil infiltration rates were also similar between MBEC and unlabeled *E. coli* injections (Fig. 6H and  Supplemental Data 3A, B). Finally, after bilateral intradermal MBEC injections, the vascular hyperemia, systemic neutrophilia, and sequential waves of myeloid and then lymphoid leukocyte infiltration were also similar to other published studies using unlabeled *E. coli* injection in humans (Fig. 6I–K and Supplementary Data 3C).^20–23^ Importantly, no adverse events were reported after intradermal MBEC injection in healthy volunteers. Hence, the addition of a MB label to the existing intradermal *E. coli* model was safe and had no effect on neutrophil function or acute inflammation.

### 3.6 MBEC can measure phagocytosis in human skin during the onset of inflammation

Having shown that (1) *E. coli* can be stably labeled with MB, (2) phagocytosis of MBEC can be measured ex vivo, and (3) MBEC can be safely injected intradermally into healthy volunteers to cause inflammation similar to after unlabeled *E. coli* injection, we next investigated whether MBEC could be used to measure phagocytosis in human skin, in vivo. Mouse models were not required because (1) numerous fluorescently labeled particles to measure phagocytosis in mouse models are already commercially available, (2) the safety of intradermal unlabeled *E. coli* injection in humans is already well established, and (3) the quantity of MB attached to killed *E. coli* was negligible compared with the doses used in clinical practice (Fig. 3E).

**Fig. 7.**
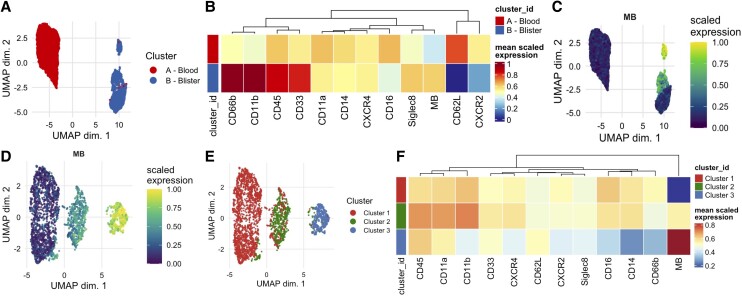
Neutrophil responses 2 to 3 h after intradermal MBEC injection in healthy volunteers. (A-C) UMAP (A), heatmap (B), and MB fluorescence (C) of blood and blister neutrophils sampled 2 to 3 h after intradermal injection of 3 × 10^7^ CFU MBEC in healthy volunteers (n = 7). (D–F) MB fluorescence (D), UMAP (E), and heatmap (F) of blister neutrophils sampled 2 to 3 h after the intradermal injection of 3 × 10^7^ CFU MBEC in healthy volunteers (n = 7).

Because neutrophils first appeared in human skin biopsies 2 to 3 h after intradermal MBEC injection (Fig. 6H), leukocytes were sampled from dermal suction blisters and the venous blood of healthy volunteers at this time point. Due to their primed CD66b^hi^CD11b^hi^CD45^hi^CD62L^lo^ expression profile, blister neutrophils clustered separately to blood neutrophils during unsupervised Uniform Manifold Approximation and Projection (UMAP) analysis. This showed that blister-derived neutrophils were an extravasated and tissue-derived phenotype, and not a hemorrhagic contaminant produced as a byproduct of the blistering process (Fig. 7A, B). In keeping with this, blister neutrophils were also MB^hi^ compared with blood neutrophils (Fig. 7B, C), demonstrating that MB accumulated in neutrophils locally at the site of MBEC injection, rather than through systemic dissemination of MB. This suggested that intradermal MBEC injection could be used to measure bacterial phagocytosis in human skin, in vivo.

To examine this in more detail, blood neutrophils were excluded from the analysis. During unsupervised UMAP analysis the remaining blister neutrophils separated into 3 clusters of increasing MB fluorescence (Fig. 7D). This again suggested that intradermal MBEC injection could be used to measure phagocytosis in human skin in vivo, and also that the transfer of MB into neutrophils was not caused by diffusion, which would otherwise cause MB to distribute equally into all blister neutrophils. MB^lo^ blister neutrophils (cluster 1) in this analysis (Fig. 7D–F) were therefore likely to be an infiltrating phenotype, that had extravasated but not yet phagocytosed MBEC. This was supported when the bacterial receptors CD11b, CD66b, CD14, and CD16 were all lower expressed in MB^hi^ blister neutrophils (cluster 3) compared with the MB^int^ (cluster 2) and/or MB^lo^ (cluster 1) blister neutrophil clusters (Fig. 7D–F), in keeping with progressive pathogen-receptor internalization during in vivo MBEC phagocytosis.^45, 46^ In summary, these data suggested that 2 to 3 h after intradermal injection (i.e. during the onset phase of acute inflammation), MBEC could be used to measure bacterial phagocytosis in human skin, in vivo.

### 3.7 MBEC can measure phagocytosis in human skin in vivo during the resolution phase of acute inflammation

**Fig. 8.**
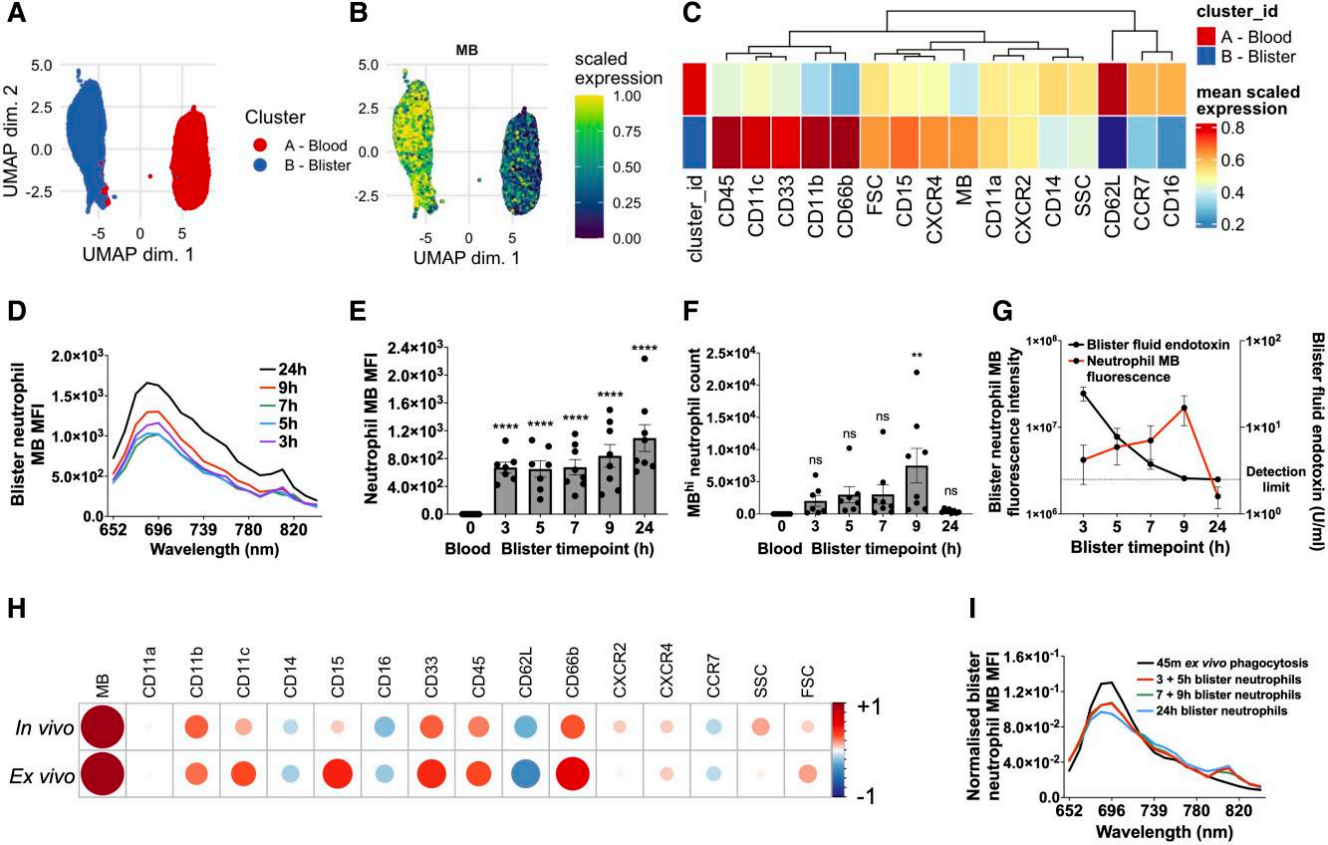
Neutrophil responses 3 to 24 h after intradermal MBEC injection in healthy volunteers. (A–C) UMAP (A), MB fluorescence (B), and heatmap (C) of blood and blister neutrophils sampled 3 to 24 h after intradermal injection of 6 × 10^7^ CFU MBEC in healthy volunteers (n = 19). (D) Red emission spectra of MB^hi^ blister neutrophils measured by spectral flow cytometry 3 to 24 h after intradermal injection of 6 × 10^7^ CFU MBEC in healthy volunteers (n = 19, 7/8 samples per time point). (E, F) MB MFI of blood and blister neutrophils (E) and MB^hi^ blister neutrophil counts (F) 3 to 24 h after intradermal injection of 6 × 10^7^ CFU MBEC in healthy volunteers (n = 19, 7/8 samples per time point). (G) MB fluorescence intensity (MB MFI × cell count) of blister neutrophils (red, left y-axis) and blister fluid endotoxin levels (black, right y-axis) from suction blisters raised 3 to 24 h after intradermal injection of 6 × 10^7^ MBEC CFU in healthy volunteers (n = 19, 7/8 samples per time point). (H) Correlations between MB and cell surface markers in blood and blister neutrophils sampled after in vivo intradermal MBEC injection (“in vivo”) and after ex vivo MBEC phagocytosis assays using circulating neutrophils (“ex vivo”) in healthy volunteers (n = 19). (I) Red emission spectra of MB^hi^ circulating neutrophils after a 45 min ex vivo MBEC phagocytosis assay, and of MB^hi^ blister neutrophils 3 to 24 h after intradermal injection of 6 × 10^7^ CFU MBEC in healthy volunteers, analyzed by spectral flow cytometry. Data were corrected for total MB MFI at each time point to allow for side-by-side comparison of neutrophil MB emission spectra between conditions. Significance reported using 1-way analysis of variance with Dunnett's multiple comparisons test (E, F). **P ≤ 0.01, ****P ≤ 0.0001. CD = cluster of differentiation; FSC = forward scatter; ns = not significant; SSC = side scatter.

We then extended this model to investigate whether MBEC could be used to measure phagocytosis in vivo in healthy volunteers at later time points after intradermal injection (i.e. at 3, 5, 7, 9, and 24 h). 19 volunteers underwent bilateral MBEC injection and suction blister formation, each person contributing to the time course dataset either (1) a 3 and 5 h blister, (2) a 7 and 9 h blister, or (3) two 24 h blisters (38 data points, n = 7/8 per time point). In keeping with inflammation onset and then resolution, the number of blister neutrophils peaked at 9 h, but by 24 h had fallen to around 1,000 cells per blister (Supplementary Data 3C). At these later phase time points, blood and blister neutrophils again clustered separately during UMAP analysis (Fig. 8A). Blister neutrophils were also MB^hi^ compared with blood neutrophils (Fig. 8B, C), even more so than in blister neutrophils sampled during inflammation onset (Fig. 7C). This was supported by visualization of the MB emission spectrum in blister neutrophils when analyzed by spectral flow cytometry (Fig. 8D), which also confirmed that neutrophil MB MFI was not caused by an increase in neutrophil autofluorescence during the extravasation process.

Further in keeping with this model being able to measure MBEC phagocytosis in vivo, the MB MFI of blister neutrophils (Fig. 8E) and MB^hi^ blister neutrophil abundance (Fig. 8F) both increased over time. In addition, the total MB fluorescence in all blister neutrophils (MB MFI × neutrophil count = MB fluorescence intensity) increased alongside the fall in blister exudate bacterial endotoxin levels (i.e. bacterial clearance from the extracellular to the intracellular space) (Fig. 8G). Together, these data suggested that after intradermal MBEC injection, the MBEC model could also identify when bacterial clearance was complete, and therefore the exact onset of inflammatory resolution.

The hypothesis that intradermal MBEC injection could be used to measure phagocytosis in humans in vivo was further supported when the statistical correlations between MB MFI and expression of the other neutrophil surface molecules were the same whether neutrophils were sampled from the in vivo MBEC model (i.e. after intradermal MBEC phagocytosis) or from an ex vivo phagocytosis assay (Fig. 8H). Put simply, the observation that both in vivo and ex vivo MBEC phagocytosis caused similar changes in neutrophil surface marker expression suggested that MB uptake in both contexts was caused by the same process (i.e. by MBEC phagocytosis).

This was further supported when the MB in 24 h blister neutrophils exhibited more red shift than blister neutrophils from earlier time points (Fig. 8I), in keeping with progressive MB phagolysosomal oxidation in infiltrating neutrophils over time (Fig. 5E). This, in turn, suggested that, in addition to bacterial phagocytosis, intradermal MBEC injection could also be used to measure phagolysosomal oxidation in human skin, in vivo.

**Fig. 9.**
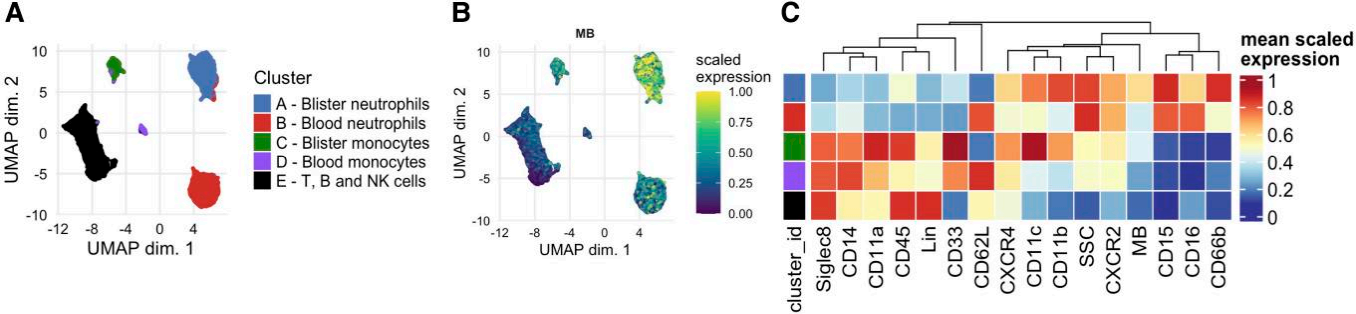
Leukocyte responses 3 to 24 h after intradermal MBEC injection in healthy volunteers. (A–C) UMAP (A), MB fluorescence (B), and heatmap (C) of blood and blister leukocytes (excluding Siglec8^hi^ eosinophils) sampled 3 to 24 h after intradermal injection of 6 × 10^7^ CFU MBEC in healthy volunteers (n = 19). NK = natural killer.

Finally, an unsupervised UMAP analysis of all blood and blister leukocyte subtypes after intradermal MBEC injection (excluding Siglec8^hi^ eosinophils) (Fig. 9A) showed that, in addition to neutrophils, blister monocytes were also MB^hi^ compared with their circulating counterparts (Fig. 9B, C), and blood and blister T, B, and natural killer cells were MB^lo^. The exclusive presence of MB in the phagocytic dermal leukocyte subsets further suggested that this new MBEC model could indeed be used to measure bacterial phagocytosis in human skin, in vivo.

Together, these data show that UV-killed *E. coli* can be labeled with MB, safely injected into the skin of healthy volunteers, and used to measure phagocytosis and phagolysosomal oxidation during both inflammation onset and its resolution.

## 4. Discussion

These results show that UV-killed *E. coli* could be labeled with MB, safely injected into the skin of healthy volunteers, and used to measure phagocytosis in human skin, in vivo. In parallel, infiltrating leukocyte abundance and surface molecule expression could be measured by detecting conjugated antibody staining alongside MB fluorescence. Finally, during phagocytosis of MBEC, phagolysosomal oxidation caused a red shift in neutrophil MB fluorescence. Hence, after a single intradermal MBEC injection, bacterial phagocytosis and phagolysosomal oxidation could, for the first time, be measured in human tissues, in vivo. This, in turn, allowed dermal MB^lo^ infiltrating neutrophils to be distinguished from MB^hi^ phagocytosing neutrophils, and also accurate identification of the end of bacterial clearance, and hence the onset of inflammatory resolution.

The majority of bacterial infections occur in epithelial tissues, primarily the skin, lungs, intestines, and genitourinary tract.^47,48^ This model is the first to measure bacterial phagocytosis in human tissues, in vivo.^44,49^ The use of MB as a bacterial stain depends on its overall positive charge, which originates from the quaternary amine in its molecular structure.^50–52^ This allows MB to bind to anions such as DNA, RNA, lipopolysaccharides, and glycoproteins, in turn explaining its nuclear staining pattern in fixed MB-labeled leukocytes (Fig. 2D).^53–55^ An alternative label to MB could be the recently developed fluorescently labeled antibiotic 7-nitrobenz-2-oxa-1,3-diazole-labeled ubiquicidin, which, albeit having a less well-established safety profile compared with MB, is also safe for human injection.^56, 57^ However, having been designed to visualize free bacteria in the human lung in vivo, its stability during phagocytosis, and therefore its response to phagolysosomal oxidation, has not been established.

The only existing model capable of measuring phagocytosis at the cellular level in humans in vivo uses synthetic skin chambers placed on top of the exposed dermis, which is uncovered by deroofing suction blisters raised on the naïve skin of healthy volunteers.^49^ In this model, neutrophils are recruited into the skin chambers using autologous serum, before fluorescein-labeled heat-killed *E. coli* are added to measure phagocytosis in extravasated neutrophils.^49^ However, being a nonresolving model, this skin chamber technique does not generate the sequential waves of neutrophils, monocytes, and T lymphocytes seen in the self-resolving MBEC model. Therefore, it cannot be used to measure monocyte phagocytosis, infer innate-adaptive immune cell interactions, or investigate the nonphagocytic roles of myeloid cells during the resolution phase of acute inflammation.^49,58–62^ Furthermore, the existing skin chamber technique measures phagocytosis in solution and on the dermal surface, and not within the skin tissue architecture.^49^ It is therefore more similar to an ex vivo phagocytosis assay using primed neutrophils than to true in vivo phagocytosis, which as mentioned is usually parenchymal. In contrast, by using bacteria as the sole inflammatory stimulus, and sampling leukocytes from within the dermal interstitium, the MBEC model is more physiological and more closely resembles an actual self-limiting bacterial skin infection.

Another disadvantage of the existing skin chamber model is that fluorescein is quenched under acidic conditions, such as those found in the maturing phagolysosome.^63, 64^ In contrast, MB is stable under acidic conditions, and during the current investigation remained fluorescent throughout MBEC phagocytosis.^65, 66^ The well-recognized photostability of MB derives from the ability of delocalized electrons in its planar aromatic rings to dissipate absorbed light energy and become stabilized by protonation (Fig. 1), as also occurs within the maturing phagolysosome.^67–71^ In keeping with this, the relatively few blister neutrophils present 24 h after intradermal MBEC injection still contained MB. Although interpreting intravital stains is increasingly challenging at later time points postinjection, this was supported by the well-recognized antiapoptotic effects of neutrophil migration into sites of inflammation, and also by the observations from a 3-dimensional ex vivo phagocytosis assay scaffold, which showed that neutrophils still contained labeled bacteria 16 h after phagocytosis.^72^

The ability of the MBEC model to measure phagolysosomal oxidation shows not only that MBEC are a fluorescently labeled particle capable of initiating inflammation and measuring phagocytosis, but also that in this context MB is a ratiometric dye sensitive to phagolysosomal oxidation. Combining a ratiometric dye with conjugated antibody staining and flow cytometry is a relatively new concept, recently described using Fluorescence Resonance Energy Transfer dyes, which are typically used to study protein-protein interactions ex vivo.^73–75^ Using a single label as both a fluorescent probe and a ratiometric dye is a useful approach in human in vivo model design, as it increases information yield (and therefore discovery potential) without the additional risk to participants of a second particle or injection.

Another unavoidable limitation of intravital staining is that the injected dye can affect the function it is designed to measure. However, in the current investigation, compared with unlabeled *E. coli*, intradermal MBEC injection did not affect the hyperemic response to bacterial injection, in vivo. Furthermore, MB did not affect ex vivo phagocytosis, at least not until the MB concentration was increased to 1 × 10^−3^% (2 orders of magnitude above that in MBEC supernatants), or until the MBEC MOI was increased to 1 × 10^3^ (3 orders of magnitude above that found in humans after intradermal UV-killed *E. coli* injection).^44^ This is supported by a recent study by Trevisan et al.,^76^ who showed that neutrophil phagocytosis of *Candida albicans* was unaffected by 1 × 10^−2^% MB. Three studies have shown that after spinal cord or lung injury in rats, MB reduces neutrophil migration into sites of inflammation.^77–79^ However, the doses of MB used in these studies was 11 orders of magnitude over those present in MBEC supernatants in our experiments. This was supported in the current investigation when neutrophil infiltration was similar between intradermal unlabeled *E. coli* and MBEC injections. Hence, although the effects of MB on leukocyte migration were not formally tested ex vivo, at the MB concentrations used in the MBEC model, MB is highly unlikely to affect neutrophil phagocytosis or infiltration in human skin, in vivo.

This new model has several important potential applications. In basic research, it could help understand how bacterial uptake is divided between the phagocyte subtypes, the role of non-phagocytic myeloid cell subtypes, and whether the previously identified differences between neutrophil and monocyte phagolysosomal oxidation ex vivo are also present in vivo.^80–83^ Furthermore, by separating early-arriving MB^lo^ “infiltrating”, MB^hi “^phagocytosing”, and late-arriving MB^lo^ “resolution-phase” phagocytes, the MBEC model could help inform studies investigating leukocyte extravasation and the noncanonical roles of phagocytic leukocytes during inflammation onset and its resolution.

In translational research, the MBEC model could help explain why some people are at higher risk of bacterial infection, such as males, the elderly, or patients with recent surgery, diabetes mellitus, or malnutrition.^84–86^ In clinical research, it could help clarify whether new or existing therapies can accelerate phagocytosis in vivo, whether defective phagocyte function is the cause of unexplained immunodeficiency, or why some therapies, such as corticosteroids, predispose to infection.^87, 88^

The current investigation could also serve as a roadmap for the development of similar human challenge models, designed to answer different experimental questions. For example, labeling *E. coli* with other clinically safe dyes (e.g. indocyanine green, rose Bengal, or acriflavine), which have their own unique fluorescent properties, and therefore potential research applications. Alternatively, labeling different bacterial species (e.g. *Staphylococcus aureus*) with MB could help investigate species-specific differences in phagocytosis and phagolysosomal oxidation in human skin, in vivo.^89–91^

In addition to those already mentioned, one limitation of the MBEC model is its incompatibility with cell membrane permeabilization, and therefore with intracellular antibody staining. This is likely due to surface and phagolysosomal membrane disruption caused by permeabilization, which results in MB dissolution. This precludes phagocytosis measurement in myeloid cell subtypes that are only identifiable by their intracellular molecule expression.^92^

Another limitation is that because naïve skin is largely devoid of neutrophils, in the MBEC model blister neutrophil MB MFI is affected by leukocyte infiltration rate.^93, 94^ Put simply, early-infiltrating or fast-moving neutrophil populations are likely to contain more MB, as they have had more time to phagocytose MBEC at a time closer to the MBEC injection, when more bacteria are present and therefore available for phagocytosis. This makes direct comparison of the phagocytic capacity of different leukocyte subtypes between time points during the inflammatory response more complex, as their MB MFI is also dependent on their rate of infiltration into, and time of arrival at, the site of MBEC injection. However, this is an unavoidable byproduct of the self-resolving and inherently physiological nature of the new MBEC model, which itself is accompanied by the advantages already described previously.

In conclusion, this study showed that the intradermal injection of methylene blue-labelled *E. coli*, followed by suction blister formation and flow cytometric analysis of infiltrating leukocytes, can measure bacterial phagocytosis and phagolysosomal oxidation in human tissues, in vivo. This new model is the first of its kind and has a number of important potential research applications that could significantly improve our understanding of the immune response to bacterial infection in humans, in vivo.

## Supplementary Material

qiae217_Supplementary_Data

## Data Availability

The data that support the findings of this study are available from the corresponding author, DG, upon reasonable request.
