# Supplementary material for: A new model measuring bacterial phagocytosis and phagolysosomal oxidation in humans using the intradermal injection of methylene blue–labeled Escherichia coli
Source: J Leukoc Biol. 2024 Oct 16;117(2):qiae217. doi: 10.1093/jleuko/qiae217 (PMC11879004; doi:10.1093/jleuko/qiae217)
Supplement: qiae217_Supplementary_Data [file qiae217_supplementary_data.zip › supplementary_figure_1.pdf]

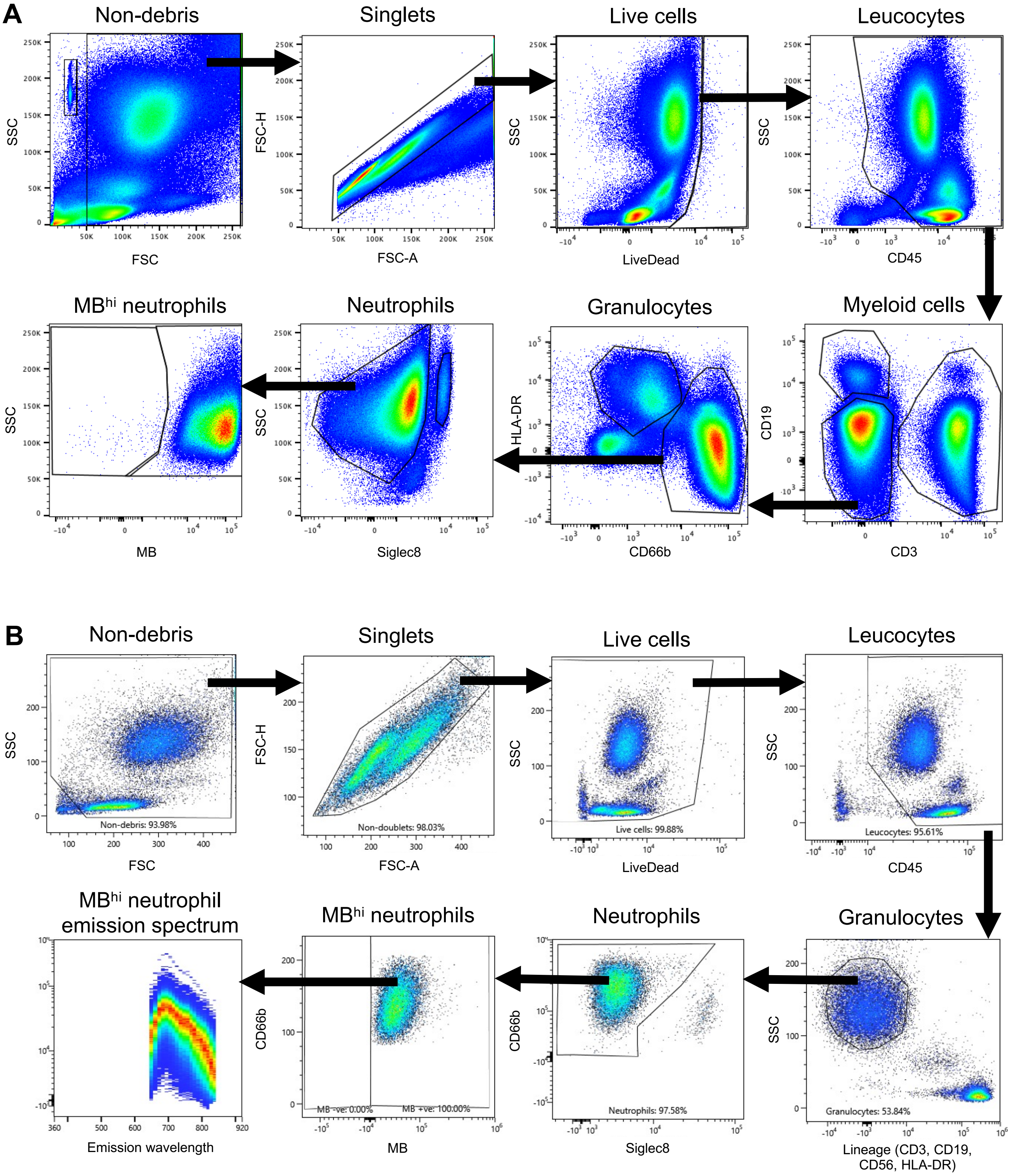

Flow cytometry gating strategies. Flow cytometry gating strategy to identify MB fluorescence in neutrophils using conventional flow cytometry **(A)** and spectral flow cytometry **(B).** Leucocytes from an *ex vivo* phagocytosis assay using MBEC (MOI = 40) were stained with the antibody panels in Section 4.8 and analysed by conventional (A) and spectral (B) flow cytometry. FSC = forward scatter, HLA = human leucocyte antigen, MB = methylene blue, Siglec = sialic-acid-binding immunoglobulin-like lectin, SSC = side scatter.
