# Supplementary material for: A new model measuring bacterial phagocytosis and phagolysosomal oxidation in humans using the intradermal injection of methylene blue–labeled Escherichia coli
Source: J Leukoc Biol. 2024 Oct 16;117(2):qiae217. doi: 10.1093/jleuko/qiae217 (PMC11879004; doi:10.1093/jleuko/qiae217)
Supplement: qiae217_Supplementary_Data [file qiae217_supplementary_data.zip › supplementary_figure_3.pdf]

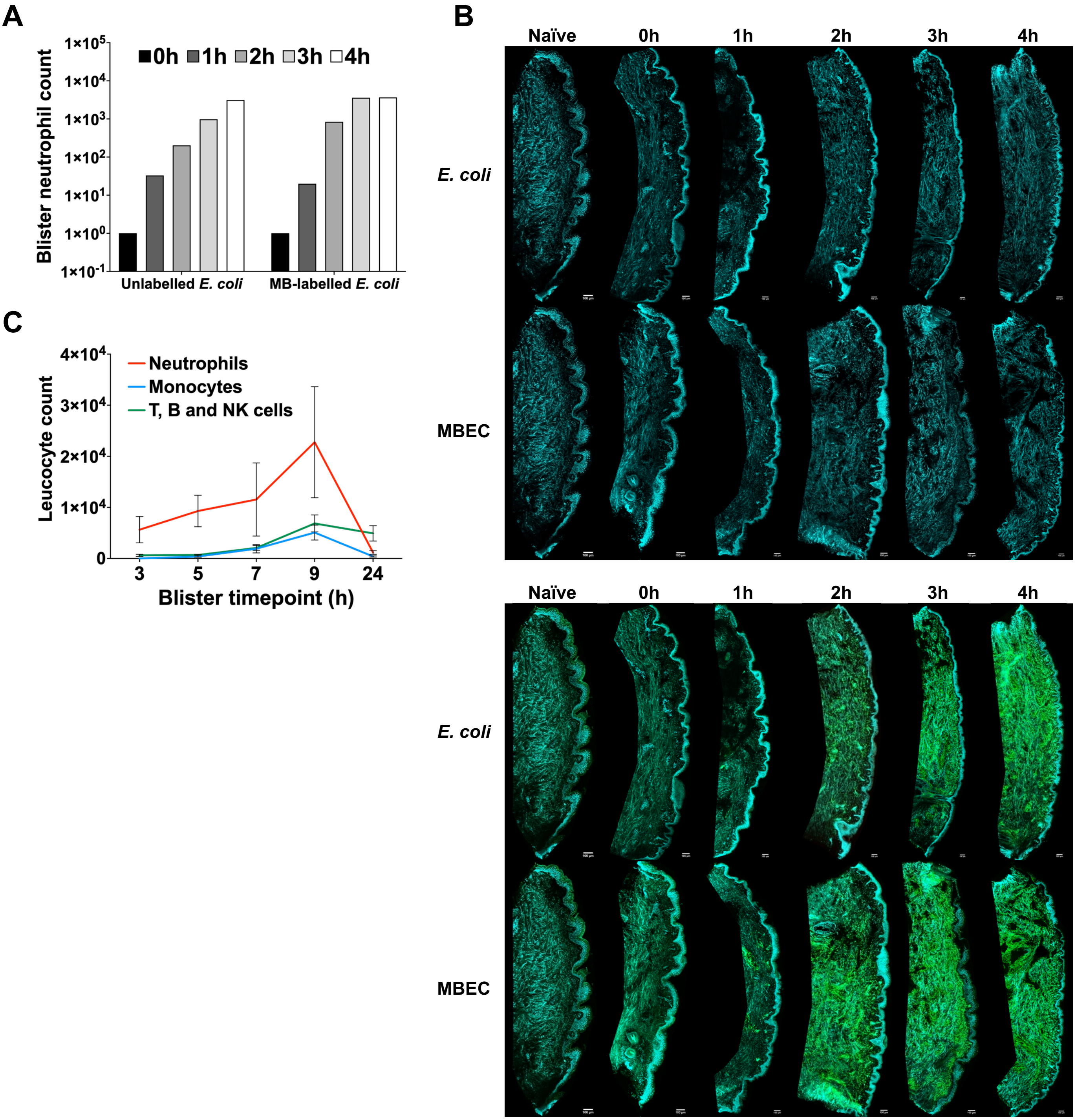

Leucocyte infiltration after intradermal MBEC injection. **(A, B)** 5 healthy volunteers underwent intradermal injection of unlabelled *E. coli* into the left volar forearm and MB-labelled *E. coli* into the right volar forearm. This was followed by either suction blister formation (A) or skin biopsy (B) at 0h, 1h, 2h, 3h, or 4h post-injection (n=1 time-point per participant). **(A)** Shown are the number of neutrophils present in blister exudates for each bacterial type at each time-point (n=1 per time-point). **(B)** Shown are the representative skin biopsy sections in Fig. 6H for each bacterial type at each time-point, except with Hoechst counterstaining (above) and merged Hoechst (blue) and FITC-CD66b (green) staining (below). **(C)** 19 healthy volunteers were injected intradermally with MBEC into the left and right volar forearms and suction blisters sampled 3h, 5h, 7h, 9h, and 24h post-injection. Shown are the absolute blister leucocyte counts for each time-point post-injection (n=19, 7/8 samples per time-point). MBEC = methylene blue-labelled *E. coli*, NK = natural killer.
